# Geography, rurality, and community distress: deaths due to suicide, alcohol-use, and drug-use among Colorado Veterans

**DOI:** 10.1186/s40621-023-00416-x

**Published:** 2023-02-10

**Authors:** Talia L. Spark, Colleen E. Reid, Rachel Sayko Adams, Alexandra L. Schneider, Jeri Forster, Lauren M. Denneson, Mary Bollinger, Lisa A. Brenner

**Affiliations:** 1 VISN 19 VA Rocky Mountain MIRECC for Veteran Suicide Prevention, Rocky Mountain Regional VA Medical Center, Veterans Health Administration, 1700 North Wheeling St., Aurora, CO 80045 USA; 2 Department of Physical Medicine and Rehabilitation, Anschutz School of Medicine, University of Colorado, Aurora, CO USA; 3 Injury and Violence Prevention Center, Anschutz School of Medicine, University of Colorado, Aurora, CO USA; 4 Geography Department, University of Colorado Boulder, Boulder, CO USA; 5 Institute for Behavioral Health, Heller School for Social Policy and Management, Brandeis University, Waltham, MA USA; 6 VA HSR&D Center to Improve Veteran Involvement in Care, VA Portland Health Care System, Portland, OR USA; 7 Department of Psychiatry, Oregon Health & Science University, Portland, OR USA; 8 VA HSR&D Center for Mental Healthcare and Outcomes Research, North Little Rock, AR USA; 9 VA HSR&D Suicide Prevention Impact Network, Little Rock, AR USA; 10 Center for Health Services Research, University of Arkansas for Medical Sciences, Little Rock, AR USA; 11 Department of Psychiatry, Anschutz School of Medicine, University of Colorado, Aurora, CO USA; 12 Department of Neurology, Anschutz School of Medicine, University of Colorado, Aurora, CO USA

**Keywords:** Suicide, Substance use disorder, Overdose, Alcohol-related liver disease, Veterans, Rurality, Geography, Community distress

## Abstract

**Background:**

In the USA, deaths due to suicide, alcohol, or drug-related causes (e.g., alcohol-related liver disease, overdose) have doubled since 2002. Veterans appear disproportionately impacted by growing trends. Limited research has been conducted regarding the relationship between community-level factors (e.g., rurality, community distress resulting from economic conditions) and the presence of spatial clustering of suicide, alcohol-related, or drug-related deaths. We explored community-level relationships in Colorado Veterans and compared suicide, alcohol-, and drug-related death rates between the Colorado adult population and Veterans.

**Methods:**

2009–2020 suicide, alcohol-related, and/or drug-related deaths were identified using qualifying multiple cause-of-death International Classification of Disease (ICD)-10 codes in CDC WONDER for the general adult population and Colorado death data for Veteran populations. Age and race adjusted rates were calculated to compare risk overall and by mortality type (i.e., suicide, alcohol-related, drug-related). In Veteran decedents, age-adjusted rates were stratified by rurality and community distress, measured by the Distressed Communities Index. Standardized mortality ratios were calculated to measure spatial autocorrelation and identify clusters using global and local Moran’s *I*, respectively.

**Results:**

6.4% of Colorado Veteran deaths (*n* = 6948) were identified as being related to suicide, alcohol, or drugs. Compared to rates in the general population of Colorado adults, Veterans had 1.8 times higher rates of such deaths overall (2.1 times higher for suicide, 1.8 times higher for alcohol-related, 1.3 times higher for drug-related). Among Veterans, community distress was associated with an increased risk of alcohol-related [age-adjusted rate per 100,000 (95% CI) = 129.6 (89.9–193.1)] and drug-related deaths [95.0 (48.6–172.0)]. This same significant association was not identified among those that died by suicide. Rurality was not associated with risk for any of the deaths of interest. There was significant spatial clustering for alcohol-related deaths in southeast Colorado.

**Conclusions:**

Colorado Veterans have higher rates of deaths due to suicide, alcohol-related, and drug-related causes compared to members of the general adult population. Upstream prevention efforts, such as community-based interventions targeting alcohol-use and community economic distress, are warranted. More research is also needed to understand how community distress and other social determinants of health impact the community burden of suicide, alcohol-related, and drug-related mortality.

**Supplementary Information:**

The online version contains supplementary material available at 10.1186/s40621-023-00416-x.

## Introduction

Mortality from suicide, alcohol-related causes (e.g., alcohol-related liver disease), or drug-related causes (e.g., overdose) has increased twofold over the past 20 years (Case and Deaton 2015; Centers for Disease Control and Prevention 2021). Most public health response has focused on the suicide and opioid epidemics (Center for Disease Control and Prevention National Center for Injury Prevention and Control. 2021; World Health Organization 2021); however, alcohol-related deaths have also doubled and require more attention (Haley et al. 2020). While often discussed individually, a growing body of research is investigating how these crises overlap and possible shared drivers including economic distress or despair (Shanahan and Copeland 2021). This research has predominantly focused on identifying individual-level factors that increase the risk of such deaths, e.g., age, race, ethnicity, gender, and educational attainment (Case and Deaton 2015; Olfson et al. 2021). However, investigating the spatial distribution of mortality and ecologic community factors could inform community-based prevention efforts. As such, more research is needed to understand how community factors can influence risk for suicide, alcohol-related, and drug-related mortality, as well as what community-based prevention efforts could be effective and where to target those efforts for the greatest impact (Ullman et al. 2021).

Available research indicates that rates of deaths due to suicide, alcohol-use, and drug-use spatially cluster, with some regions or counties having higher rates than others (Dwyer-Lindgren et al. 2018; Khana et al. 2018; Rossen et al. 2013). This spatial distribution is likely tied to how other risk and protective factors also cluster in space. Along those lines, some researchers have used ecological-level data (e.g., county, ZIP code, or census tract) to measure associations between suicide, alcohol-related, and drug-related mortality with factors such as rurality and community distress (i.e., deprivation from poor economic conditions). For example, residing in rural communities is associated with increased risk of suicide, alcohol-related, and drug-related mortality collectively and suicide and drug overdose, specifically (Monnat 2020; Rossen et al. 2018; Rossen et al. 2013; Searles et al. 2014). Additionally, studies using different measures of community economic distress suggest that deaths due to suicide, drug overdose, and alcohol-related deaths are higher in communities with more distress (Knapp et al. 2019; Monnat et al. 2019; Steelesmith et al. 2019). For example, counties with higher eviction rates also have higher alcohol- and drug-related mortality rates (Bradford and Bradford 2020).

Of note, Veterans appear to be disproportionately impacted by increases in suicide, alcohol-related, and drug-related mortality (Katz et al. 2020; Peltzman et al. 2020; VA Office of Mental Health and Suicide Prevention 2021a). While the increased risk of suicide among Veterans is well-documented (VA Office of Mental Health and Suicide Prevention 2021a), less is known about the increased risk of alcohol-related and drug-related mortality among Veterans. Additionally, limited research has been conducted regarding the impact of rurality or other community factors on such deaths among Veterans. Nonetheless, work to date suggests that rural-residing Veterans enrolled in Veteran Health Administration (VHA) care are at greater risk for death by suicide when compared to urban-residing Veterans (McCarthy et al. 2012; Shiner et al. 2020).

Using a population of Colorado Veteran decedents, the objective of this study was to: (1) understand how Veteran rates of suicide, alcohol-related, and drug-related deaths compared to rates in the general adult population; and (2) explore similarities and differences in Veteran mortality rates by rurality and community distress, as well as the spatial clustering of these deaths in a population.

## Methods

### Data sources

Death certificate data for all Veteran deaths 2009–2020 were provided by the Colorado Department of Public Health and Environment Vital Statistics Program. Data for each death included the year of death, age, gender, race, ethnicity, marital status, county of residence, underlying cause of death, and up to 11 multiple cause-of-death codes in the International Classification of Disease Version 10 (ICD-10) format. Age-specific counts of suicide, alcohol, and drug-related deaths in all Colorado adults (≥ 18 years) from 2009–2020 were retrieved from the Centers for Disease Control and Prevention WONDER Multiple Cause of Death database (2021).

Population estimates for Veterans by county were derived from the American Community Survey (ACS) 5-year Veteran Population Estimates, from 2012, 2014, and 2016 with breakdowns by age-groups (18–34, 35–54, 55–64, 65–74, and 75 +).

### Measures

#### Outcome

Suicide, alcohol-related, and drug-related deaths were identified using all available multiple cause-of-death fields provided in death certificates. Any death with at least one qualifying ICD-10 code present was identified using a case definition developed in a previous analysis (Spark et al. 2022). Qualifying ICD-10 codes included deaths due to suicide; unintentional and undetermined alcohol poisonings and drug overdoses; alcohol- and drug-related chronic disease; and mental and behavioral health codes indicating substance use disorder. Deaths were labeled as suicide, alcohol-related, or drug-related based on qualifying codes, see the supplemental material for specific classification (Additional file 1: eTable 1). For most analyses, individual decedents could be present in multiple categories of mortality type, for example, if the suicide involved alcohol or a drug, or if multiple qualifying codes were present (e.g., polysubstance overdose). For the descriptive comparison by individual-level factors, individuals included in multiple mortality type categories were included in a > 1 mortality type.

#### Time period

The year of death was categorized as overall (2009–2020) or as two 6-year periods (2009–2014 and 2015–2020). Given the small numbers at the county level, this was the smallest year grouping that limited data suppression.

#### Rurality

Rurality was assigned at the county level using definitions outlined by the Colorado Rural Health Center (Colorado Rural Health Center 2021). Counties designated as Metropolitan Areas were categorized “urban,” those with a population density of 6 or fewer persons per square mile were “frontier,” and the remaining counties “rural.”

#### Distressed communities index

The Distressed Communities Index (DCI) developed by the Economic Innovation Group (Fikri and Lettieri 2018), was used to quantify economic distress at the county level. The DCI uses seven census bureau measures (i.e., no high school diploma, housing vacancy rate, unemployment rate, poverty rate, median income ratio, change in employment, and changes in business establishments) to score and stratify all US counties into quintiles defined as “prosperous,” “comfortable,” “mid-tier,” “at risk,” and “distressed.” Prior research has identified a correlation between distress, as measured by DCI, and poorer health outcomes (Charles et al. 2019; Hawkins et al. 2020).

### Approach/analysis

To compare Veteran and general population rates of suicide, alcohol-related, and drug-related mortality (Aim 1), direct age- and race-adjusted rates and 95% confidence intervals (CI) were calculated using the 2000 US population as the standard population and the normal approximation to calculate CI (Curtin and Klein 1995). As needed, age and race groups were collapsed because of small sample sizes (*n* < 5). Rates were calculated for qualifying deaths overall and by mortality type (i.e., suicide, alcohol-related, drug-related) for the Colorado general adult and Veteran populations. Population estimates provided by the Centers for Disease Control and Prevention Wide-ranging Online Data for Epidemiologic Research (CDC WONDER) were used for the denominator of the adult population, while the midpoint Veteran population (2015) from ACS was used for Veteran rate estimates.

Next, to address the second aim, descriptive statistics comparing distributions of qualifying Veteran deaths overall and by mutually exclusive mortality type for demographic variables (i.e., year of death, age at death, gender, race, ethnicity, marital status, rurality of county, and DCI) were calculated. Chi-squared values were calculated to compare the distribution of demographic variables across mortality types.

Next, age-adjusted rates for Veteran deaths were calculated and stratified by rurality, and DCI by mortality type. Finally, for qualifying Veteran deaths overall, age-adjusted rates and 95% CIs were stratified by both rurality and DCI; with this double-stratification, counts were too small to explore by mortality type. Adjustment by race was not possible given small sample sizes even after collapsing age and race categories.

Because of small numbers at the county level, all county-level rates were presented as Standardized Mortality Ratios (SMR), calculated using indirect adjustment (Curtin and Klein 1995). Here statewide Veteran age-specific rates were used as the standard population and the midpoint Veteran populations as the denominator. Choropleth maps were used to visualize county-level SMR, DCI, and rurality. Global Moran’s *I*, a summary measure of spatial autocorrelation that describes the overall spatial clustering of a single variable, was calculated overall and by mortality type for all three time periods to identify measures that were spatially dependent (Anselin et al. 2007). Monte Carlo simulation (*n* = 1000) was used to calculate *p* values. Any measure with a significant *p* value indicating spatial dependence was further investigated using local Moran’s *I*, which specifies where spatial clustering is occurring (Anselin 1995). Local Moran’s *I* results were visualized to show which counties with high SMRs neighbored counties with high SMRs (high–high). Neighbor weight matrices were defined using a distance-based weight matrix using the county centroid. A threshold distance of 64.7 miles was identified so that each county had at least two neighbors. Secondary analysis was done using the queen contiguity neighbor structure.

R version 4.0.5 (R Statistical Programming, Vienna, Austria) was used for analyses and bar chart figures. SMRs were calculated using the epitools package (Aragon 2020). Neighbor weight matrices, global, and local Moran’s *I* were calculated using the spdep package (Bivand 2006). Choropleth maps were visualized using ArcGIS Pro (Version 2.6.0, Esri, Inc.). *p* values < 0.05 and non-overlapping 95% CIs were considered significant. Counts or rates calculated from counts < 10 were suppressed. This research was approved as exempt by the VA Office of Research & Development, Colorado Multiple Institutional Review Board, and CDPHE Institutional Review Board.

## Results

Of the 109,314 Veterans dying in Colorado 2009–2020, 6948 (6.4%) were identified as having a qualifying multiple cause-of-death ICD-10 code indicating suicide, alcohol-related, or drug-related mortality. Comparing the age- and race-adjusted Veteran rates to the rates in the full Colorado adult population, Veterans had 1.8 times higher rates of such deaths overall and significantly higher rates for each mortality type: 1.3 times higher for drug-related deaths (37.2 vs. 28.2 per 100,000), 1.8 times higher for alcohol-related deaths (81.4 vs. 44.2 per 100,000), and 2.1 times higher for suicide deaths (52.3 vs. 24.4 per 100,000) (Table 1).

**Table 1:** Comparison of suicide, alcohol-related, and drug-related mortality rates between all Colorado residents at 18 + and Colorado Veterans, 2009–2020

Mortality type	Colorado adult population^a^		Colorado Veteran population^b^		Rate ratio^d^
	Number of deaths	Age- and race-adjusted rate 95% CI per 100,000	Number of deaths^c^	Age- and race-adjusted rate 95% CI per 100,000	
Overall	43,220	85.8 (84.9–86.6)	6885	153.7 (149.5–158.0)	1.8
Suicide^e^	12,643	24.4 (23.9–24.8)	2336	52.3 (49.9–54.8)	2.1
Alcohol-related	22,239	44.2 (43.6–44.9)	3805	81.4 (78.4–84.5)	1.8
Drug-related^e^	14,423	28.2 (27.7–28.7)	1403	37.2 (35.1–39.4)	1.3

^a^Counts taken from CDC Wonder 2009–2020 Multiple Cause-of-Death data, age-adjusted rates calculated using 2015 American Community Survey Population estimates as denominator and 2000 US census population as the standard population

^b^Counts provided by Colorado Department of Public Health and Environment, Vital Statistics Program. Age-adjusted rates calculated using 2015 American Community Survey Population estimates as denominator and 2000 US census population as the standard population

^c^Veterans with unknown or missing race (*n* = 63) were excluded from this analysis to calculate race adjustment

^d^Rate ratio compares age-adjusted rate in Colorado Veteran Population to Colorado Adult Population

^e^AI/AN, Asian, and Black groups combined for age- and race-adjusted rates since cell sizes were < 5 for Asian/Pacific Islander (API), American Indian/Alaskan Native (AIAN) and Black aged 65 + (suicide) and API and AI/AN 65+  (drug)

Of all the Veteran suicide, alcohol, and drug-related deaths, the majority (49%, *n* = 3439) were categorized as alcohol-related only, 28% (1,969) were categorized as suicides only, 13% (922) drug-related only, and 9% (618) had more than one mortality type identified (Table 2). The distribution of qualifying deaths was higher in 2015–2020 as compared to 2009–2014 across all mortality types, with no significant differences between types. The demographic distribution of mortality types significantly differed by sex, age, race, ethnicity, and marital status. Of note, the proportion of drug-related deaths was higher in Black Veterans compared to other mortality types, alcohol-related deaths were higher in American Indian or Alaska Native Veterans versus other mortality types, and both alcohol- and drug-related deaths were higher in Hispanic Veterans compared to suicide deaths. There were significant differences by county distress. A higher proportion of drug-related deaths occurred in prosperous counties, while a higher proportion of alcohol-related deaths occurred in distressed counties. There was no significant difference by rurality.

**Table 2:** Colorado Veteran population characteristics by mortality type, 2009–2020

Variable	Overall (*n* = 6948)	Suicide only (*n* = 1969)	Alcohol-related only (*n* = 3439)	Drug-related only (*n* = 922)	> 1 mortality type^a^ (*n* = 618)	*p* value^b^
Year						0.196
2009–2014	3218 (46.3)	915 (46.5)	1624 (47.2)	400 (43.4)	279 (45.1)	
2015–2020	3730 (53.7)	1054 (53.5)	1815 (52.8)	522 (56.6)	339 (54.9)	
Sex						** < 0.001**
Female	275 (4.0)	56 (2.8)	107 (3.1)	70 (7.6)	42 (6.8)	
Male	6673 (96.0)	1913 (97.2)	3332 (96.9)	852 (92.4)	576 (93.2)	
Age						** < 0.001**
18–34	628 (9.0)	382 (19.4)	56 (1.6)	130 (14.1)	60 (9.7)	
35–54	1682 (24.2)	499 (25.3)	610 (17.7)	312 (33.8)	261 (42.2)	
55–64	1,928 (27.7)	320 (16.3)	1164 (33.8)	284 (30.8)	160 (25.9)	
65–74	1,603 (23.1)	337 (17.1)	1,044 (30.4)	133 (14.4)	89 (14.4)	
75 +	1,107 (15.9)	431 (21.9)	565 (16.4)	63 (6.8)	48 (7.8)	
Race						** < 0.001**
Asian American or Pacific Islander	48 (0.7)	21 (1.1)	15 (0.4)	6 (0.7)	6 (1.0)	
American Indian or Alaska Native	90 (1.3)	16 (0.8)	58 (1.7)	7 (0.8)	9 (1.5)	
Black or African-American	376 (5.4)	55 (2.8)	206 (6.0)	86 (9.3)	29 (4.7)	
White	6371 (91.7)	1866 (94.8)	3124 (90.8)	813 (88.2)	568 (91.9)	
Other and unknown	63 (0.9)	11 (0.6)	36 (1.0)	10 (1.1)	6 (1.0)	
Ethnicity						** < 0.001**
Hispanic	854 (12.3)	137 (7.0)	548 (15.9)	108 (11.7)	61 (9.9)	
Non-Hispanic	6042 (87.0)	1815 (92.2)	2874 (83.6)	801 (86.9)	552 (89.3)	
Unknown	52 (0.7)	17 (0.9)	17 (0.5)	13 (1.4)	5 (0.8)	
Marital status						** < 0.001**
Single/never married	1195 (17.2)	350 (17.8)	512 (14.9)	207 (22.5)	126 (20.5)	
Married	2520 (36.4)	894 (45.6)	1129 (32.9)	302 (32.9)	195 (31.7)	
Divorced	2510 (36.2)	501 (25.5)	1413 (41.2)	360 (39.2)	236 (38.3)	
Widowed	625 (9.0)	208 (10.6)	323 (9.4)	42 (4.6)	52 (8.4)	
Unknown	78 (1.1)	9 (0.5)	54 (1.6)	8 (0.9)	7 (1.1)	
Rurality						0.09
Frontier	207 (3.0)	62 (3.1)	114 (3.3)	20 (2.2)	11 (1.8)	
Rural	803 (11.6)	248 (12.6)	393 (11.4)	97 (10.5)	65 (10.5)	
Urban	5938 (85.5)	1659 (84.3)	2932 (85.3)	805 (87.3)	542 (87.7)	
Distressed communities index						** < 0.0001**
Prosperous	5508 (79.3)	1556 (79.0)	2678 (77.9)	752 (81.6)	522 (84.5)	
Comfortable	194 (2.8)	60 (3.0)	100 (2.9)	17 (1.8)	17 (2.8)	
Mid-tier	485 (7.0)	142 (7.2)	254 (7.4)	49 (5.3)	40 (6.5)	
At risk	556 (8.0)	169 (8.6)	284 (8.3)	79 (8.6)	24 (3.9)	
Distressed	205 (3.0)	42 (2.1)	123 (3.6)	25 (2.7)	15 (2.4)	

*p* value <0.05 was considered significant and bolded

^a^Individuals with multiple cause-of-death ICD-10 codes that qualify as more than 1 mortality type included here

^b^*p* value results from a chi-squared test comparing all despair types

### Geography of community factors

**Fig. 1:**
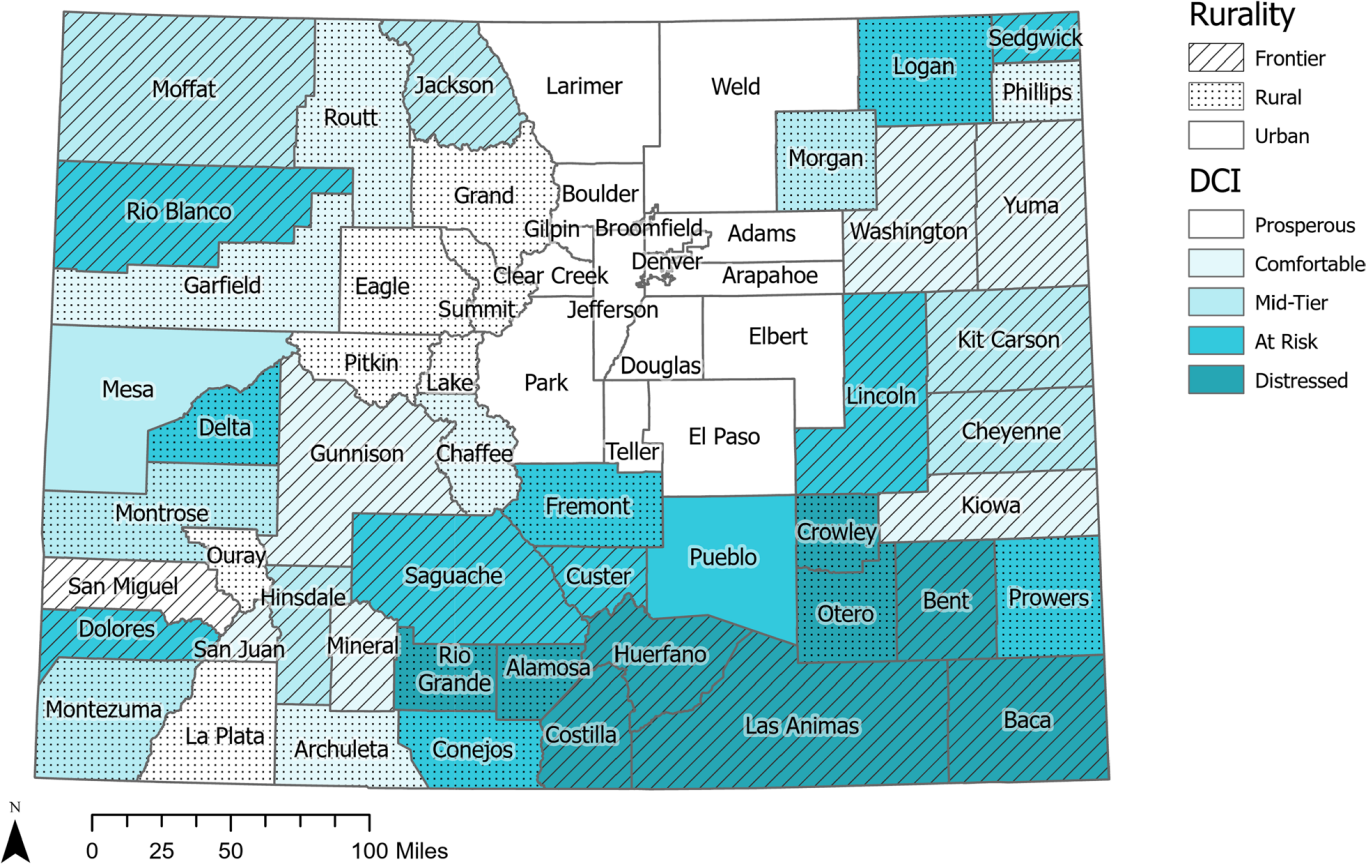
Rurality and Distressed Communities Index Categories by County in Colorado

Most urban counties in Colorado, except Mesa County along the Western border, are located along the Front Range of the Rocky Mountains, where 80% of the state’s population resides (Fig. 1). There is a mixture of rural and frontier counties in the mountainous west of the state and the eastern plains. Most urban counties are categorized as prosperous, while there are no urban counties that are considered comfortable or distressed. Additionally, there are several rural and frontier counties in Colorado categorized as prosperous or comfortable because of wealthy mountain towns such as Aspen (Pitkin County), Telluride (San Miguel County), and Vail (Summit County). The only distressed counties in Colorado are nine neighboring counties in the southeast corner of the state.

### Stratified rates by county variables

**Fig. 2:**
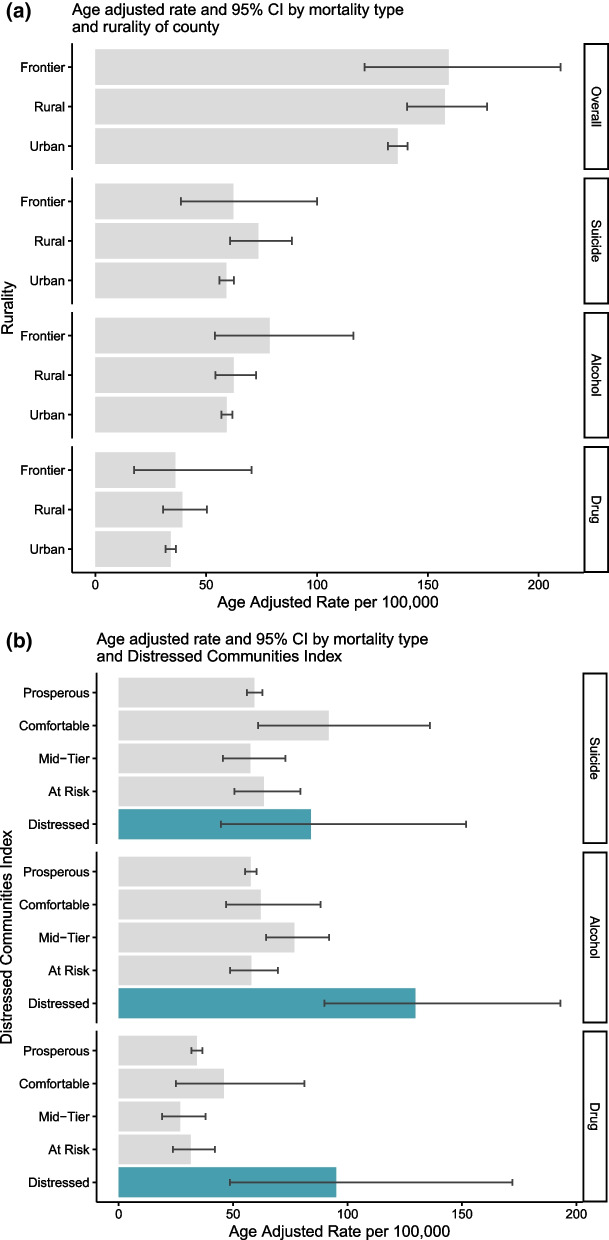

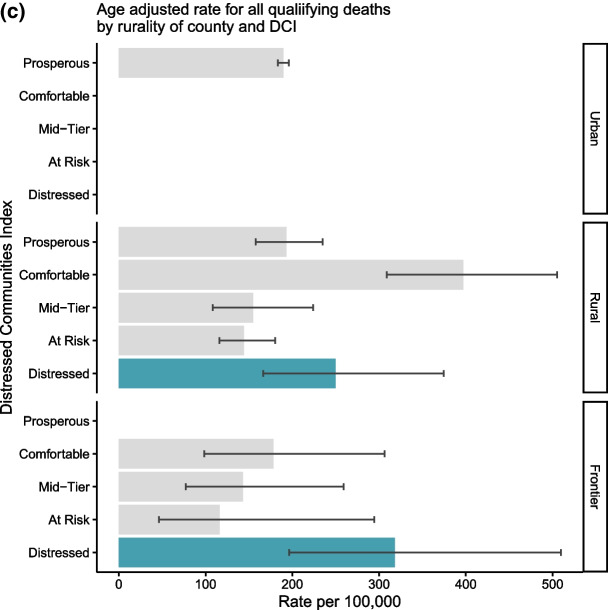
**a**–**c** Age-Adjusted Mortality Rate for Veterans by Mortality Type Stratified by **a** Rurality,* **b** Distressed Community Index, and **c** Both Rurality and Distressed Community Index for All Qualifying Deaths.**, *Rurality defined as Urban, if metro area within county, frontier if the population was ≤ 6 per square mile, and otherwise rural. ** Categories without a value had 0 or 1 qualifying counties and were suppressed

Age-adjusted rates for qualifying mortality (overall and by mortality type) did not significantly differ by rurality (Fig. 2a); however, there were significant differences by the DCI (Fig. 2b). For all qualifying deaths, distressed counties had the highest rates (age-adjusted rate (95% CI) = 275.4 (203.7–371.8) per 100,000), two times higher than prosperous counties (134.7 (130.2–139.3) per 100,000). There was not a significant difference by DCI for suicide mortality rates, though comfortable and distressed counties had the highest rates (91.7 (60.9–136.0) and 84.0 (44.7–151.8) per 100,000, respectively). For both alcohol-related and drug-related deaths, distressed counties had at least twofold higher mortality rates compared to all other groups (129.6 (89.9–193.1) for alcohol-related and 95.0 (48.6–172.0) for drug-related deaths)), while prosperous, comfortable, mid-tier, and at-risk counties had similar mortality rates.

Finally, we investigated the stratification of distressed counties by rurality (Fig. 2c) for suicide, alcohol-related, and drug-related deaths overall. Wide confidence intervals and some categories having ≤ 1 qualifying county precluded identifying many significant differences; therefore, we describe possible trends. Comfortable rural counties had the highest age-adjusted qualifying mortality rates per 100,000 followed by distressed frontier counties. Meanwhile, frontier counties with other DCI classifications had lower age-adjusted rates than comparable rural counties with overlapping CIs. Most urban counties were prosperous with similar rates compared to rural and frontier prosperous counties. There were no urban counties in Colorado that were defined as comfortable or distressed, and only one county was defined as mid-tier or at risk.

### Spatial analysis

County SMRs were calculated by mortality type for the full time period (Additional file 1: eFigure 1). Using the age-specific Veteran mortality rate for the full state and the standard population for SMR calculations, there was 1 county that had a significantly higher than expected number of deaths (or significant SMR) for all qualifying deaths, Denver County, which was also significantly higher for alcohol-related and drug-related deaths.

Within global Moran’s *I* results (Additional file 1: eTable 2) for all mortality types over the full time period and split into 6-year groupings, only alcohol-related deaths exhibited significant spatial autocorrelation. All other mortality types did not exhibit significant spatial autocorrelation, indicating that the SMRs did not greatly differ across the state. Global Moran’s *I* values for alcohol-related deaths were significant for the full time period and the years 2015–2020, but not 2009–2020 indicating there was a spatial pattern to how alcohol-related mortality increased in this population in more recent years.

**Fig. 3:**
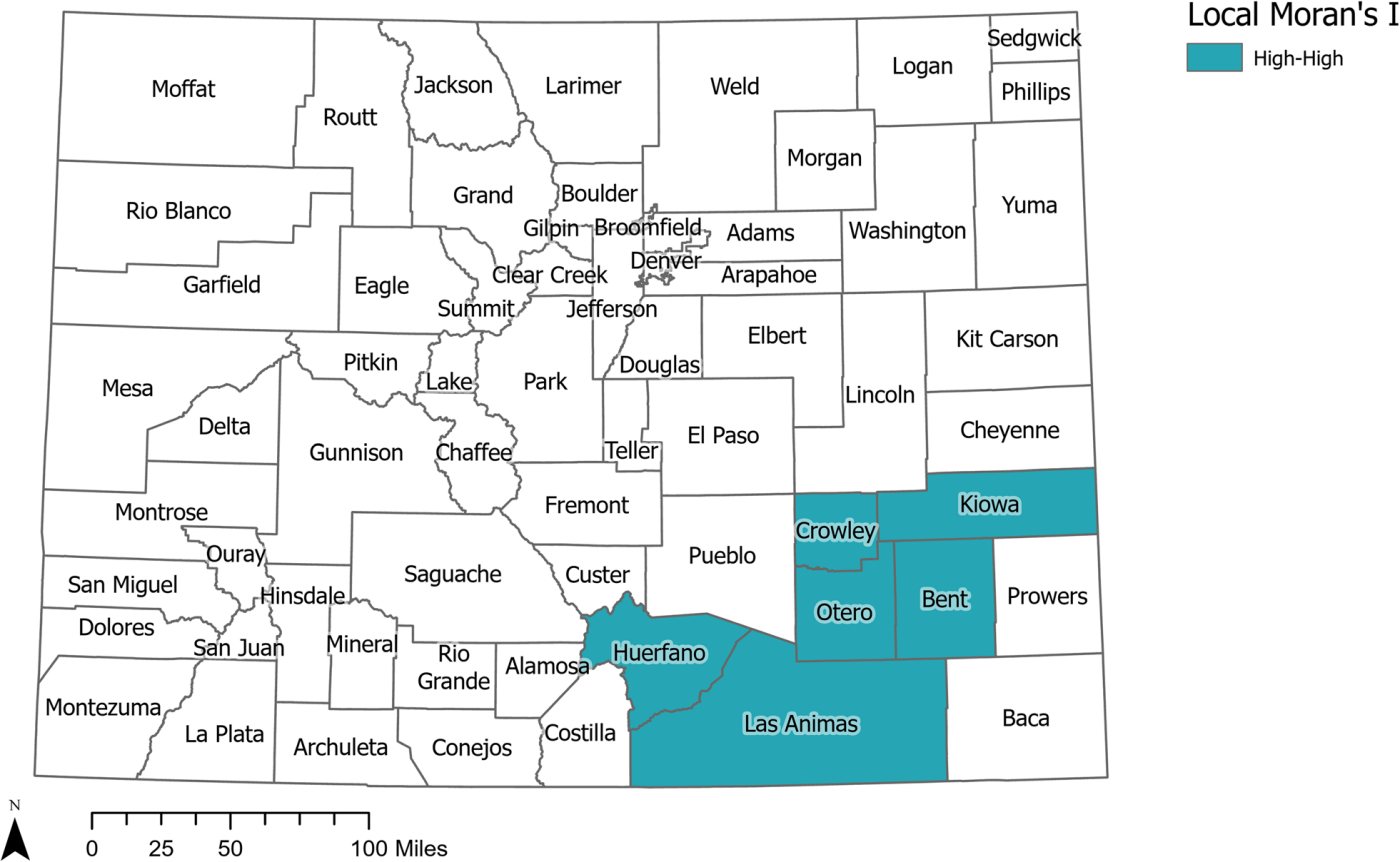
Map of Local Moran’s I Result for Veteran Alcohol-Related Deaths 2009–2020 Indicating Significant SMR Cluster. Counties Indicated as High–High have a Higher SMR with Neighboring Counties also Having Higher SMR

Local Moran’s *I* test was run only for alcohol-related mortality given the significant global Moran’s *I*. Alcohol deaths in Veterans were clustered (i.e., counties with higher SMRs neighbored counties with higher SMRs) in seven counties in the southeast corner of Colorado (i.e., Bent, Crowley, Huerfano, Kiowa, Las Animas, and Otero Counties) (Fig. 3). Only Otero, Bent, and Kiowa Counties were included in the cluster when the analysis was limited to 2015–2020 deaths. Mineral and Denver counties had higher SMRs but were neighbored by counties with lower SMRs. The majority (58%) of Veteran deaths included in the 2009–2020 cluster of high alcohol-related deaths had an indication of an alcohol-use disorder, while 35% of deaths had an indication of physical ailments related to alcohol-use (e.g., alcohol-related liver disease) and 8% alcohol poisoning. Sensitivity analysis using a different weight matrix did not drastically change results, indicating the stability of findings.

## Discussion

We explored the spatial clustering of suicide, alcohol-related, and drug-related mortality and the relationship between these deaths and community factors (i.e., rurality and community distress) in a population of 2009–2020 Colorado Veteran decedents. We found that the risk of all qualifying deaths was 80% higher for Veterans compared to the rate in the general population of Colorado adults, with a 114% higher risk of suicide, 84% higher risk of alcohol-related deaths, and 31% higher risk of drug-related deaths. Additionally, community distress, but not rurality, was associated with the risk of qualifying deaths, with the most distressed counties having the highest rates of alcohol-related and drug-related deaths among Veterans. Further, when stratifying suicide, alcohol-related, and drug-related mortality rates by both rurality and community distress, the highest risk DCI category differed by rurality with rural “comfortable” counties having the highest risk for suicide, alcohol-related, and drug-related mortality followed by “distressed” rural and frontier counties. Meanwhile, a spatial cluster of alcohol-related deaths was identified in very rural Southeastern Colorado. Taken together, these findings indicate a complicated relationship between the spatial distribution of rurality and community economic distress and impacts to the risk of suicide or alcohol-related or drug-related mortality among Colorado Veterans.

Suicide, alcohol-related, and drug-related mortality rates in Veterans, while significantly higher than the general population, did not differ by rurality. Moreover, suicide and drug-related deaths were uniform across the state with limited variation in standardized mortality ratios. These findings were unexpected given that abundant research in the general population indicates that suicide and overdose rates are associated with rurality and spatially cluster in the general population (Rossen et al. 2018; Rossen et al. 2013; Searles et al. 2014). These results also do not align with previous findings indicating suicide risk in Veterans is around 20% higher in rural communities (McCarthy et al. 2012; Shiner et al. 2020). However, given that previous research has predominantly focused on Veteran populations enrolled in VHA care, and VHA-accessing populations differ from non-VHA-accessing populations in important ways (Wong et al. 2016), our findings could be more consistent with actual risk in Veterans. Alternatively, it is unclear if these findings only represent Colorado Veterans or can be generalized to Veterans in other states. More research is needed in a national Veteran population to understand if: Veteran suicide, alcohol-related, and drug-related risk for death is consistently higher than non-Veterans; there is spatial variation between such death and location within states; whether rurality is an important risk factor; and, if risk varies among Veterans who are using the VHA versus those not using VHA.

As mentioned, unlike Veteran suicide and drug-related deaths, alcohol-related deaths had significant spatial variation, with a spatial cluster of mainly alcohol-use disorder or alcohol liver disease mortality among Veterans in Southeast Colorado. Of note, this cluster corresponded with some of the most remote Colorado counties with the highest levels of community distress. While there are large community- and clinic-based programs within the VA targeting opioid overdose and suicide prevention (Oliva et al. 2017; VA Office of Mental Health and Suicide Prevention 2021b), increased community-based responses to alcohol-use disorder are warranted. More broadly, targeted economic support in these counties could also help address both upstream and downstream impacts of distress.

Among members of the Veteran population, differences in alcohol-related and drug-related mortality risk by community distress were notable, with distressed counties having nearly two times the risk of communities with other DCI levels. Moreover, the finding of possible modification of the association of community distress by rurality indicates the need for further investigation. Specifically, “comfortable” rural counties had the highest rates of qualifying deaths overall, while “distressed” counties had the highest rates within frontier counties, though these were not significantly higher due to wide confidence intervals. Another study found that in the general population distressed communities in rural counties had the highest rates of qualifying deaths (Monnat 2019). In sum, these findings indicate that rurality-focused research likely does not capture the diversity of rural communities, as well as factors associated with suicide, alcohol-related, and drug-related mortality. Rurality is often treated dichotomously as a proxy for many factors (e.g., social determinants of health, economic distress, access to resources, and cultural and social factors such as firearm culture or social isolation) (Cromartie and Bucholtz 2008). Instead, economic concerns in a county are distinct from rurality designation; thus, not all rural communities experience these concerns to the same extent. Our findings indicate a complicated picture exists, and studies using more complex methods conducted among larger cohorts are needed to better understand how dimensions of community factors interact to influence the risk of behavioral health-related morbidity and mortality.

While not the focus of this analysis, differences in individual-level demographic distributions by mortality type were identified, notably by age, race, and ethnicity. Some populations (e.g., American Indian or Alaska Native, Hispanic) had a higher proportion of alcohol-related or drug-related deaths compared to suicides, so it is possible that risk for these mortality outcomes differentially impacts specific demographic populations, resulting in varying mortality-related outcomes from the same exposure. Of interest, Liu et al. (2017) looked at suicide risk in relation to interactions between neighborhood composition, ethnicity, income, and socially disadvantaged propositions and found that income comparison can have negative consequences, thereby highlighting the importance of combining sociodemographic and community-level data.

There are several important limitations. First, our sample might not generalize to all Veterans given that it was conducted in Colorado. Specific to opioid overdose, Colorado deaths have been lower than the national average and have not followed the same timeline for national increases (Demont et al. 2022). For example, fentanyl-related deaths started increasing in 2017 in Colorado compared to 2013 nationally (Center for Disease Control and Prevention National Center for Injury Prevention and Control 2021; Demont et al. 2022). Additionally, the Veteran cohort only included people indicated as having served in the Armed Forces. This field on the death certificate could capture individuals currently serving in the Armed Forces and simultaneously, misclassify Veterans as having not served. Therefore, these results might not represent all Colorado Veterans, though a study conducted using 2004–2008 Colorado death data found this field to be 92% accurate (Bahraini et al. 2012). Also, Colorado is a coroner state where death investigators are elected and might not have training in medicine or forensics, so these results could differ from states with a medical examiner or combined coroner and medical examiner systems (Fierro 2003). There are other possible contributors to measurement error of exposure and outcome. There are several standardized approaches to measure both rurality and community distress that are based on different data and methodologies. While these measures generally correlate, using different definitions could alter some of our findings. Additionally, deaths due to suicide, alcohol-use, and drug-use are likely underreported on death certificates due to misclassification or exclusion of relevant codes (Castle et al. 2014; Snowdon and Choi 2020). Therefore, the true burden is likely higher than what we report. Finally, findings could have been impacted by small numbers given that suicide and substance-related mortality are rare events. For example, we were not able to adjust for more than age in some analyses due to small cell sizes, while additionally adjusting for race, ethnicity, and sex is preferable given demographic differences in risk. We addressed small numbers by collapsing multiple years, demographic categories for adjustment, and conducting analyses at the county level. This approach could have missed important phenomena occurring at a smaller geographic scale or time periods. Small numbers could have also limited our ability to find spatial differences. To address some of these limitations, analyses should be repeated in a national sample of Veterans. Also, small area estimate techniques could be incorporated to better account for small numbers and scale (Khana et al. 2018). Finally, multiple measures of rurality and community distress should be used to probe the robustness of findings.

## Conclusion

Community and clinic-based interventions are increasingly being used by the VA to prevent suicide (VA Office of Mental Health and Suicide Prevention 2021b) and opioid overdose (Oliva et al. 2017). Given the higher risk of suicide and drug-related mortality in Veterans, these are important efforts; however, with alcohol-related deaths being a higher contributor to mortality, increased focus on the prevention of alcohol-related deaths is warranted. Additionally, more research is needed to identify which communities are most at risk, how community risk influences individual risk, and how community-based prevention could improve outcomes. This study is among the first to examine how community factors can contribute to Veteran risk of suicide, alcohol-related, and drug-related mortality and how such deaths spatially cluster. We found that community distress was associated with the risk of alcohol and drug-related deaths in Colorado Veterans. Current trends in suicide, alcohol-related, and drug-related deaths represent a complex web of historical, political, economic, and demographic trends with differential impacts on individuals and communities (Friedman et al. 2020). More work into how these factors predict excess mortality, as well as societal or community-based interventions to prevent such death, is needed.

## Supplementary Information

**Additional file 1.** **Figure S1**: Map of Veteran Suicide, Alcohol-Related, and Drug-Related Standardized Mortality Ratio (SMR) by county for 2009-2020. Counties with mortality counts < 10 were suppressed. **Table S1**: Qualifying Codes by Mortality Type. **Table S2**: Global Moran’s I Results for County SMR by Qualifying Veteran Deaths Overall and by Mortality Type (i.e., Suicide, Alcohol-Related, and Drug-Related) and Year. Significant Moran’s I indicated significant spatial dependence of variable. **Table S3**: CDC Wonder Query Results by Age for Colorado general adult population, 2009–2020.

## Data Availability

Multiple datasets were used in this study. The datasets generated from CDC during the current study are available in the Multiple Cause of Death 1999–2020 repository, available at https://wonder.cdc.gov/mcd-icd10.html. Specific queries and age-specific data are provided in the supplemental material (Additional file 1: eTable 3). The Colorado Veteran death data generated during and/or analyzed during the current study are not publicly available due to Colorado state guidelines around confidentiality. Data can be requested through the Colorado Department of Public Health and Environment request system (https://www.datarequest.dphe.state.co.us/) and may require Institutional Review Board approval.

## Abbreviations

ACS: American Community Survey
CDC WONDER: Centers for Disease Control and Prevention’s Wide-ranging Online Data for Epidemiologic Research
CI: Confidence intervals
DCI: Distressed Communities Index
ICD-10: International Classification of Disease Version 10
SMR: Standardized mortality ratio
VA: Veteran Affairs
VHA: Veteran Health Administration
