# Supplementary material for: Geography, rurality, and community distress: deaths due to suicide, alcohol-use, and drug-use among Colorado Veterans
Source: Inj Epidemiol. 2023 Feb 10;10:8. doi: 10.1186/s40621-023-00416-x (PMC9912586; doi:10.1186/s40621-023-00416-x)
Supplement: Supplementary file 1 — Additional file 1. Figure S1: Map of Veteran Suicide, Alcohol-Related, and Drug-Related Standardized Mortality Ratio (SMR) by county for 2009-2020. Counties with mortality counts < 10 were suppressed. Table S1: Qualifying Codes by Mortality Type. Table S2: Global Moran’s I Results for County SMR by Qualifying Veteran Deaths Overall and by Mortality Type (i.e., Suicide, Alcohol-Related, and Drug-Related) and Year. Significant Moran’s I indicated significant spatial dependence of variable. Table S3: CDC Wonder Query Results by Age for Colorado general adult population, 2009–2020. [file 40621_2023_416_MOESM1_ESM.docx]

eTable 1: Qualifying Codes by Mortality Type

| Despair Type | ICD 10 Codes^a^ |
| --- | --- |
| Suicide | X60 X61 X62 X63 X64 X65 X66 X67 X68 X69 X70 X71 X72 X73 X74 X75 X76 X77 X78 X79 X80 X81 X82 X83 X84 Y87.0 |
| Alcohol-related | E24.4 F10.0 F10.1 F10.2 F10.3 F10.4 F10.5 F10.6 F10.7 F10.8 F10.9 G31.2 G62.1 G72.1 I42.6 K29.2 K70.0 K70.1 K70.2 K70.3 K70.4 K70.9 K85.2 K86.0 R78.0 T51.0 X45 X65 Y15 |
| Drug-related | F11.0 F11.1 F11.2 F11.3 F11.4 F11.5 F11.7 F11.8 F11.9 F12.0 F12.1 F12.2 F12.3 F12.4 F12.5 F12.7 F12.8 F12.9 F13.0 F13.1 F13.2 F13.3 F13.4 F13.5 F13.7 F13.8 F13.9 F14.0 F14.1 F14.2 F14.3 F14.4 F14.5 F14.7 F14.8 F14.9 F15.0 F15.1 F15.2 F15.3 F15.4 F15.5 F15.7 F15.8 F15.9 F16.0 F16.1 F16.2 F16.3 F16.4 F16.5 F16.7 F16.8 F16.9 F18.0 F18.1 F18.2 F18.3 F18.4 F18.5 F18.7 F18.8 F18.9 F19.0 F19.1 F19.2 F19.3 F19.4 F19.5 F19.7 F19.8 F19.9 G21.1 G44.4 J70.2 J70.3 J70.4 K85.3 M87.1 R50.2 R78.1 R78.2 R78.3 R78.4 R78.5 T40.0 T40.1 T40.2 T40.3 T40.4 T40.5 T40.6 T40.7 T40.8 T40.9 T42.3 T42.4 T42.6 T43.6 X40 X41 X42 X43 X44 X60 X61 X62 X64 X85 Y10 Y11 Y12 Y13 Y14 |

*eFigure 1: Map of Veteran Suicide, Alcohol-Related and Drug-Related Standardized Mortality Ratio (SMR) by county for 2009-2020. Counties with mortality counts <10 were suppressed.*


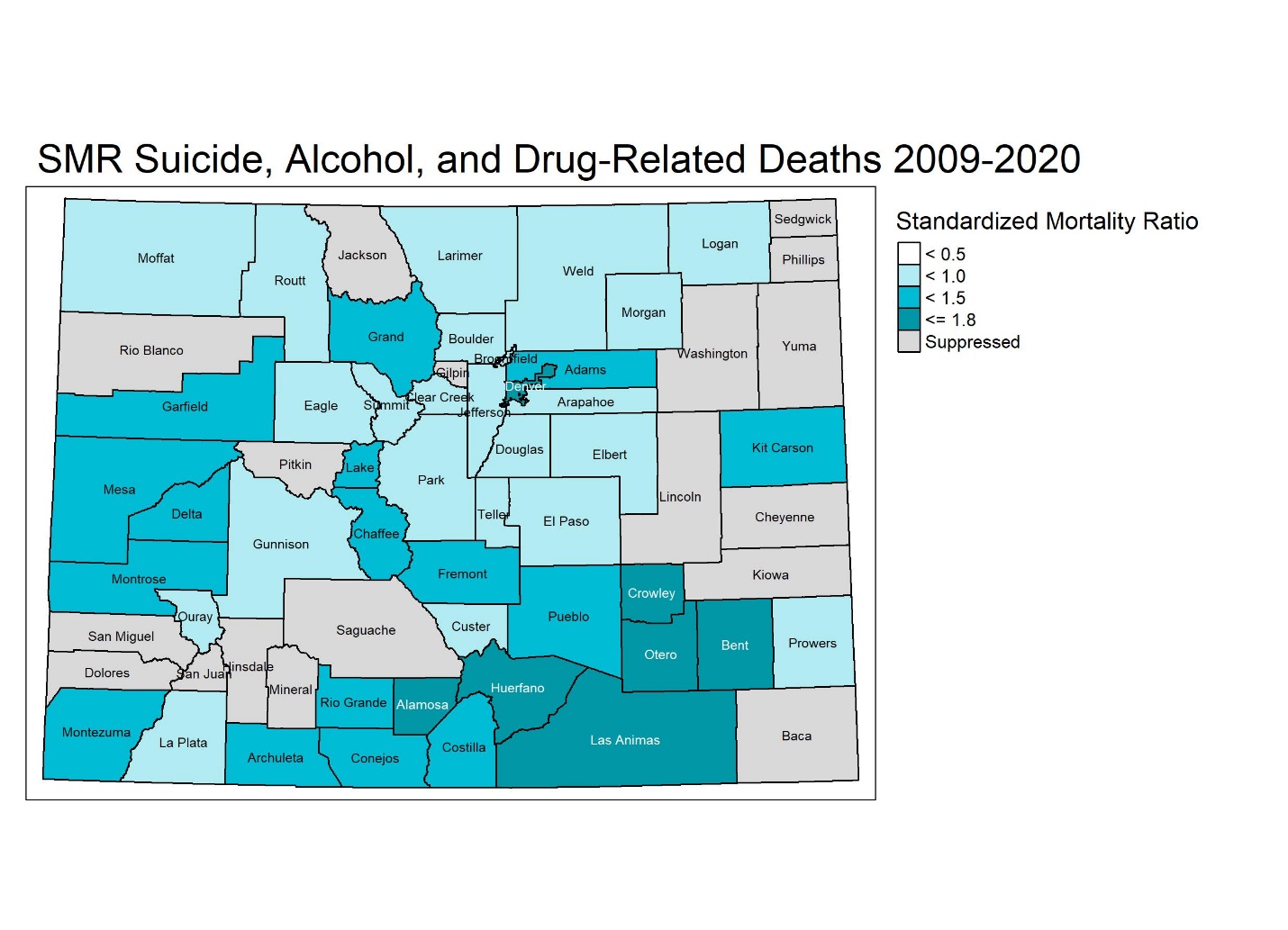


eTable 2: Global Moran’s I Results for County SMR by Qualifying Veteran Deaths Overall and by Mortality Type (i.e., Suicide, Alcohol-Related, and Drug-Related) and Year. Significant Moran’s I indicated significant spatial dependence of variable.

|  | | At Least 2 Neighbors | | Queen Contiguity | |
| --- | --- | --- | --- | --- | --- |
| Mortality Type | Year | Moran's I | P-value | Moran's I | P-value |
| Overall | 2009-2020 | 0.081 | 0.08 | 0.037 | 0.22 |
| Overall | 2009-2014 | 0.028 | 0.23 | 0.014 | 0.34 |
| Despair | 2015-2020 | 0.070 | 0.10 | 0.038 | 0.20 |
| Suicide | 2009-2020 | -0.012 | 0.42 | -0.055 | 0.69 |
| Suicide | 2009-2014 | -0.003 | 0.37 | -0.041 | 0.58 |
| Suicide | 2015-2020 | -0.030 | 0.52 | -0.062 | 0.72 |
| **Alcohol-related** | **2009-2020** | **0.126** | **0.02** | **0.121** | **0.04** |
| Alcohol-related | 2009-2014 | 0.082 | 0.07 | 0.094 | 0.07 |
| **Alcohol-related** | **2015-2020** | **0.112** | **0.03** | **0.115** | **0.04** |
| Drug-related | 2009-2020 | -0.005 | 0.43 | 0.003 | 0.40 |
| Drug-related | 2009-2014 | -0.007 | 0.45 | 0.022 | 0.31 |
| Drug-related | 2015-2020 | -0.038 | 0.62 | -0.047 | 0.66 |

*eTable 3: CDC Wonder Query Results by Age for Colorado general adult population, 2009-2020.*

| Age | Population | Overall^a^ | Suicide^b^ | Alcohol-related^c^ | Drug-related^d^ |
| --- | --- | --- | --- | --- | --- |
| 18 | 846,547 | 225 | 151 | 35 | 72 |
| 19 | 868,010 | 300 | 177 | 42 | 131 |
| 20 | 849,869 | 320 | 181 | 60 | 134 |
| 21 | 859,455 | 370 | 203 | 69 | 163 |
| 22 | 892,204 | 442 | 231 | 98 | 203 |
| 23 | 920,956 | 474 | 229 | 95 | 228 |
| 24 | 949,057 | 503 | 253 | 107 | 222 |
| 25 | 972,120 | 518 | 233 | 113 | 272 |
| 26 | 987,945 | 565 | 247 | 146 | 296 |
| 27 | 998,810 | 611 | 230 | 174 | 310 |
| 28 | 999,864 | 638 | 251 | 181 | 323 |
| 29 | 997,666 | 608 | 237 | 174 | 298 |
| 30 | 992,225 | 633 | 228 | 206 | 310 |
| 31 | 974,860 | 623 | 250 | 182 | 296 |
| 32 | 970,052 | 626 | 223 | 229 | 314 |
| 33 | 955,746 | 638 | 240 | 225 | 290 |
| 34 | 946,716 | 698 | 227 | 256 | 338 |
| 35 | 931,941 | 630 | 224 | 226 | 310 |
| 36 | 915,738 | 673 | 227 | 271 | 313 |
| 37 | 907,298 | 624 | 214 | 250 | 266 |
| 38 | 899,340 | 637 | 200 | 247 | 298 |
| 39 | 897,252 | 720 | 266 | 291 | 314 |
| 40 | 883,359 | 705 | 229 | 299 | 303 |
| 41 | 872,053 | 701 | 241 | 311 | 268 |
| 42 | 861,561 | 743 | 221 | 313 | 328 |
| 43 | 854,771 | 726 | 204 | 361 | 295 |
| 44 | 852,194 | 747 | 228 | 355 | 299 |
| 45 | 856,263 | 802 | 225 | 405 | 326 |
| 46 | 855,839 | 851 | 236 | 458 | 315 |
| 47 | 862,816 | 926 | 273 | 492 | 314 |
| 48 | 866,049 | 976 | 258 | 501 | 364 |
| 49 | 871,376 | 1,030 | 266 | 575 | 354 |
| 50 | 868,403 | 959 | 230 | 535 | 332 |
| 51 | 865,565 | 1,049 | 263 | 632 | 324 |
| 52 | 863,900 | 1,109 | 259 | 666 | 349 |
| 53 | 864,880 | 1,136 | 267 | 704 | 322 |
| 54 | 869,758 | 1,168 | 272 | 709 | 348 |
| 55 | 868,451 | 1,079 | 222 | 662 | 330 |
| 56 | 862,829 | 1,159 | 256 | 719 | 343 |
| 57 | 851,550 | 1,214 | 279 | 739 | 334 |
| 58 | 838,610 | 1,153 | 254 | 726 | 330 |
| 59 | 820,773 | 1,144 | 236 | 752 | 306 |
| 60 | 799,740 | 1,043 | 221 | 689 | 233 |
| 61 | 777,005 | 1,041 | 204 | 706 | 238 |
| 62 | 761,252 | 923 | 171 | 621 | 219 |
| 63 | 722,747 | 870 | 162 | 607 | 169 |
| 64 | 692,557 | 784 | 142 | 531 | 175 |
| 65 | 659,475 | 785 | 140 | 551 | 170 |
| 66 | 626,731 | 631 | 127 | 434 | 120 |
| 67 | 590,571 | 600 | 133 | 413 | 103 |
| 68 | 554,582 | 484 | 109 | 328 | 83 |
| 69 | 518,886 | 469 | 93 | 326 | 87 |
| 70 | 484,264 | 400 | 86 | 279 | 62 |
| 71 | 449,732 | 396 | 95 | 271 | 55 |
| 72 | 415,282 | 370 | 97 | 230 | 67 |
| 73 | 381,977 | 317 | 97 | 191 | 44 |
| 74 | 345,705 | 289 | 73 | 199 | 37 |
| 75 | 321,413 | 248 | 69 | 157 | 32 |
| 76 | 299,133 | 233 | 63 | 147 | 35 |
| 77 | 277,725 | 179 | 58 | 108 | 18 |
| 78 | 256,269 | 204 | 68 | 120 | 26 |
| 79 | 238,016 | 177 | 53 | 109 | 30 |
| 80 | 222,141 | 181 | 69 | 97 | 20 |
| 81 | 207,156 | 149 | 62 | 74 | 24 |
| 82 | 191,283 | 137 | 54 | 65 | 25 |
| 83 | 176,986 | 124 | 44 | 74 | 14 |
| 84 | 163,006 | 105 | 42 | 52 | 19 |
| 85+ | 974358 | 628 | 270 | 269 | 133 |
| Total | **49,852,663** | **43,220** | **12,643** | **22,239** | **14,423** |

^a^ Query available at: <https://wonder.cdc.gov/controller/saved/D77/D288F827>

^b^ Query available at: <https://wonder.cdc.gov/controller/saved/D77/D288F857>

^c^ Query available at: <https://wonder.cdc.gov/controller/saved/D77/D275F294>

^d^ Query available at: <https://wonder.cdc.gov/controller/saved/D77/D275F297>
